# mTHPC-Loaded Extracellular Vesicles Significantly Improve mTHPC Diffusion and Photodynamic Activity in Preclinical Models

**DOI:** 10.3390/pharmaceutics12070676

**Published:** 2020-07-17

**Authors:** Marie Millard, Solène Posty, Max Piffoux, Jordane Jasniewski, Henri-Pierre Lassalle, Ilya Yakavets, Florence Gazeau, Claire Wilhelm, Amanda K. A. Silva, Lina Bezdetnaya

**Affiliations:** 1 Centre de Recherche en Automatique de Nancy, Centre National de la Recherche Scientifique UMR 7039, Université de Lorraine, 54506 Vandœuvre-lès-Nancy, France; marie.millard@live.fr (M.M.); soleneposty@orange.fr (S.P.); h.lassalle@nancy.unicancer.fr (H.-P.L.); i.yakavets@gmail.com (I.Y.); 2 Research Department, Institut de Cancérologie de Lorraine, 54519 Vandoeuvre-lès-Nancy, France; 3 Laboratoire Matière et systèmes complexes, CNRS UMR 7057, Université de Paris, 75013 Paris, France; max.piffoux@cri-paris.org (M.P.); florence.gazeau@univ-paris-diderot.fr (F.G.); claire.wilhelm@univ-paris-diderot.fr (C.W.); amanda.brun@univ-paris-diderot.fr (A.K.A.S.); 4 LIBIo, Université de Lorraine, 54501 Vandœuvre-lès-Nancy, France; jordane.jasniewski@univ-lorraine.fr

**Keywords:** extracellular vesicles, liposomes, photodynamic therapy, mTHPC, grafted tumor, nanocarriers

## Abstract

Extracellular vesicles (EVs), derived from the cell, display a phospholipid bilayer membrane that protects the cargo molecules from degradation and contributes to increasing their stability in the bloodstream and tumor targeting. EVs are interesting in regard to the delivery of photosensitizers (PSs) used in the photodynamic therapy (PDT), as they allow us to overcome the limitations observed with liposomes. In fact, liposomal formulation of meta-tetra(hydroxyphenyl)chlorin (mTHPC) (Foslip^®^), one of the most potent clinically approved PSs, is rapidly destroyed in circulation, thus decreasing in vivo PDT efficacy. mTHPC-EV uptake was evaluated in vitro in a 3D human colon HT-29 microtumor and in vivo study was performed in HT-29 xenografted mice. The obtained data were compared with Foslip^®^. After intravenous injection of the mTHPC formulations, biodistribution, pharmacokinetics and PDT-induced tumor regrowth were evaluated. In a 3D model of cells, mTHPC-EV uptake featured a deeper penetration after 24h incubation compared to liposomal mTHPC. In vivo results showed a considerable improvement of 33% tumor cure with PDT treatment applied 24h after injection, while 0% was observed after Foslip^®^/PDT. Moreover, 47 days were required to obtain ten times the initial tumor volume after mTHPC-EVs/PDT compared to 30 days for liposomal mTHPC. In conclusion, compared to Foslip^®^, mTHPC-EVs improved mTHPC biodistribution and PDT efficacy in vivo. We deduced that a major determinant factor for the improved in vivo PDT efficacy is the deep mTHPC intratumor penetration.

## 1. Introduction

Photodynamic therapy (PDT) has a recognized efficacy in oncology, particularly in the treatment of small superficial tumors. This minimally invasive treatment modality is based on the combined action of the photosensitizer (PS) activated by visible light and molecular oxygen. Following irradiation, the PS generates reactive oxygen species leading to cell death and tissue destruction [1]. The efficiency of PDT with different photosensitizers, including meta-tetra-(hydroxyphenyl)chlorin (mTHPC), is still restricted by a moderate tumor selectivity of PSs, that is, elevated skin sensitivity after irradiation, thus reducing its clinical application. In order to overcome these limitations, a nanovectorization strategy with phospholipidic structures was developed and proposed for enhanced PDT [2].

Being non-immunogenic and biodegradable, liposomes are the most popular mTHPC nanocarriers studied to date [3]. However, a lack of stability with a quick redistribution of mTHPC on plasma proteins along with 60% of liposome destruction after 24 h is an obvious shortcoming [4]. This destruction is mostly due to the loss of membrane integrity [5]. In addition, it was demonstrated that Foslip^®^ (mTHPC in conventional liposomes) is rapidly removed from the bloodstream due to its strong recognition by the reticuloendothelial system and fast clearance [6].

Consequently, Foslip^®^–PDT was not much more efficient than mTHPC-based PDT. Recently, extracellular vesicles (EVs) have proved to be perspective nanocarriers for PS to overcome limitations observed with liposomes. Their membrane is composed of a phospholipid bilayer that protects the cargo molecule from degradation and contributes to increasing their natural stability in blood circulation [5,6]. Loaded with mTHPC, EVs showed enhanced drug uptake in cancer cells and reduction in cancer cell metabolism compared with liposomal and free mTHPC [7]. In 3D models of cells that mimic in vivo situations, mTHPC-EVs showed increased accumulation, diffusion and photocytotoxicity compared to liposomal and free mTHPC [5]. Recently, EVs loaded with mTHPC and magnetic nanoparticles have also demonstrated improved PDT efficacy in vivo in a murine model after intratumoral injection when compared with the free drug [8]. However, currently, no data regarding the biodistribution, pharmacokinetics and PDT efficacy after systemic injection of mTHPC-EVs have been reported in the literature.

In the present paper, we have conducted research on the photochemical properties of mTHPC-EVs and their behavior in murine plasma. We studied the distribution of these nanocarriers in a 3D model to gain a better insight into the mechanistic aspect related to the in vivo situation. We further evaluated the biodistribution, pharmacokinetics, and tumor regrowth after PDT treatment with intravenous mTHPC-EV injection. All investigated parameters were compared with the liposomal mTHPC formulation, Foslip^®^.

## 2. Materials and Methods

### 2.1. Photosensitizers

The liposomal formulation of mTHPC [3,3′,3”,3”’-(2,3-dihydroporphyrin-5,10,15,20-tetrayl) tetraphenol] (Foslip^®^) was kindly provided by biolitec research GmbH (Jena, Germany). Foslip^®^ is based on dipalmitoylphosphatidylcholine (DPPC) and dipalmitoylphosphatidylglycerol (DPPG) and mTHPC with drug:lipid ratio of 1:12 (mol/mol) and DPPC:DPPG ratio of 9:1 (*w/w*). Foslip^®^ powder was reconstituted in water for injection to obtain a stock solution at 1 mM mTHPC.

### 2.2. Animals, Tumor Model and Cell Culture

All experiments were performed in accordance with animal care guidelines from the European Union and were approved by the appropriate authority (directive 2010/63/EU, 22 September 2010). The animal projects registered under the numbers (#2438; #1353) received a favorable agreement from the Ethics Committee and were approved by the French Higher Education and Research Minister. Studies (26 October 2015) were performed using female immunodeficient Naval Medical Research Institute (NMRI^nu/nu^) mice (Janvier Labs, St Berthevin, France), aged 7 weeks. Mice were housed in standard conditions (12 h light/dark cycle, 25 °C, 50% relative humidity).

Tumors were generated by subcutaneous injection with 8 × 10^6^ exponentially growing human colon adenocarcinoma cells (HT-29) (ATCC^®^, LGC Promochem, Molsheim, France) in 5% glucose solution into the right flank or between the scapula of the mice for biodistribution experiments. Cells were grown at 37 °C (5% CO_2_, humidified atmosphere) in phenol red-free Roswall Park Memorial Institute (RPMI)-1640 medium (Invitrogen, Cergy-Pontoise, France) supplemented with 9% fetal calf serum (FCS) and 1% 200 mM L-glutamine (Life Technologies, Carlsbad, CA, USA). Photosensitizer was administrated intravenously by a tail vein injection at a dose of 0.30 mg/kg of mTHPC. Following intravenous injection, mice were kept in the dark for 96 h and experiments were realized with minimal ambient light.

### 2.3. Production of mTHPC-Extracellular Vesicles

#### 2.3.1. Human Umbilical Vascular Endothelial (HUVEC) Cells Culture in Bioreactor

Cytodex 1 dextran microcarriers (GE Healthcare) of 200 µm were dispersed in phosphate-buffered saline (PBS), and autoclaved to ensure sterility. PBS medium was changed to Dulbecco’s Modified Eagle’s (DMEM) medium without phenol red (Gibco^TM^, ThermoFisher, Waltham, MA, USA) and kept at 4 °C until further use. Before cell seeding, microcarriers were incubated in complete medium (37 °C) at a 6 g/L for at least 3 h to ensure the media oxygenation. HUVEC were cultured at 37 °C (5% CO_2_, humidified atmosphere) in DMEM supplemented with 9% heat-inactivated fetal calf serum (FCS) and 100 U/mL of penicillin and streptomycin. Cells were seeded with a cell to microcarrier ratio 5/1 and submitted to 24 cycles of 45 min of rest followed by 3 min of gentle mixing (30 to 60 rotation per minute (rpm) depending on bioreactor volume) to ensure homogeneous adhesion of cells on microcarriers. After cell adhesion, microcarriers were diluted to 3 g/L with complete medium and cells were submitted to gentle mixing (30 to 60 rpm depending on bioreactor volume) until reaching confluence on microcarriers (about 7 days) [9]. Every 2–3 days, 30 to 70% of the medium was changed depending on the cell confluence.

#### 2.3.2. Turbulence-Triggered EV Production and Loading

Turbulence-triggered EV production and loading was carried out in 1 L bioreactor by replacing the complete medium by 400 mL of serum-free DMEM medium containing 100 µM of free mTHPC. Spinner flask bioreactors were then submitted to rotation, creating a turbulent flow featuring a Kolmogorov length (smallest vortex size) of 35 µM during 4 h [10]. After that, the supernatant was collected and submitted to purification for EV isolation.

#### 2.3.3. EV Purification

EVs were washed and isolated from the conditioned culture medium with a differential (ultra)centrifugation method based on the previously described protocol by Théry et al. [11]. First, cell debris were eliminated by 2000 g centrifugation for 10 min. The total population of EVs (containing both microvesicles and exosomes) was isolated in a single 100,000 g step for 1 h. mTHPC-EVs were resuspended in serum-free medium and characterized by nanoparticle tracking analysis (NTA 3.2 Software, Malvern Instruments, Malvern, UK). mTHPC concentration was estimated by LS55 spectrofluorometer (Perkin Elmer, Waltham, MA, USA).

### 2.4. Characterization of mTHPC-EVs

#### 2.4.1. mTHPC-EV Size and Particle Concentration Measurements

Size distribution profiles and stability of mTHPC-EVs were obtained using the NanoSight LM10-T14 system (Malvern Instruments, Malvern, UK) equipped with a 532 nm laser at 50 mW and an scientific Complementary metal-oxide-semiconductor (sCMOS) camera. Before measurements, mTHPC-EVs were diluted in sterile PBS (1:5000). For each sample, five movies of 30 s were recorded using a camera level of 16 with a temperature regulated at 25 °C. Particle diameter was calculated from Stokes–Einstein equation and analyzed with nanoparticle tracking analysis (NTA) 3.2 software.

#### 2.4.2. mTHPC-EV Size and Morphology by Transmission Electronic Microscopy (TEM)

To visualize the shape and confirm the size distribution of mTHPC-EVs, cryo-TEM experiments were performed as follows: mTHPC-EVs were diluted to an appropriate concentration (1:5) in sterile PBS. An amount of 5 µL of the mTHPC-EV sample was transferred to a quantifoil^®^ (Quantifoil MicroTools GmbH, Jena Germany) membrane constituted of carbon. We then removed the liquid in excess using a filter paper and we rapidly immersed the membrane into liquid ethane which was cooled in liquid nitrogen. The samples were analyzed at the temperature of −180 °C via a LaB6 JEM2100 cryo transmission electron microscope (JEOL, Akishima, Tokyo, Japan). This equipment operates at 80 kV with a JEOL minimum dose system (MDS).

#### 2.4.3. mTHPC Quantification inside EVs

The concentration of mTHPC in purified EVs was determined by fluorescence spectroscopy. Samples were excited at 416 nm, and fluorescence was registered at 652 nm. mTHPC-EVs were diluted in sterile PBS supplemented with Triton X-100 at 0.3% final concentration in order to lyse EVs and trigger the release of mTHPC. Drug concentration was determined by using a fluorescence calibration curve obtained from a standard mTHPC solution.

The measurements of fluorescence polarization and photoinduced fluorescence quenching were conducted with LS55B spectrofluorometer (PerkinElmer, Waltham, MA, USA) equipped with polarizers, thermostated cuvette compartments, and magnetic stirring, as previously described [5,12]. The excitation wavelengths were 435 nm for fluorescence polarization and at 416 nm for photoinduced fluorescence quenching measurements, while mTHPC fluorescence was registered at 652 nm.

### 2.5. Pharmacokinetics and Biodistribution

At pre-determined time points after photosensitizer injection, mice were sacrificed when the diameter of tumors reached 6–7 mm (4–7 mice per group). Various tissue samples of the tumor, skin, muscle, spleen, liver, kidneys, heart, lungs and plasma were collected and washed in 0.9% NaCl before being frozen at −80 °C.

#### 2.5.1. Blood Sample Preparation and Mthpc Quantification

Blood was collected by intracardiac puncture and placed in heparin-coated Vacutainer^®^ blood collection tubes (BD Diagnostics, Le Pont de Claix Cedex, France). Samples were centrifuged (15 min, 1200 g at 4 °C) before analysis by spectrofluorometer to determined mTHPC concentration using a fluorescence calibration curve obtained from a standard mTHPC solution. Plasma was diluted in PBS supplemented with 1% neutral detergent Triton^®^X-100 (Sigma-Aldrich, St. Louis, MO, USA) prior to the analysis.

#### 2.5.2. Fluorescence Imaging of Mthpc Biodistribution Followed by Chemical Extraction

At each pre-determined time after injection, the excised tissues excluding plasma were analyzed by the FluorVivo^TM^ 300 image system (Indec Systems, Santa Clara, CA, USA) equipped with an LED at 510–550 nm and a long wave emission filter (630–690 nm). Fluorescence imaging data were analyzed using ImageJ software and normalized to the fluorescence intensity of control mice (PBS) before chemical extraction. Tissues were pounded in an extraction buffer containing 80% absolute ethanol, 20% DMSO and 1% acid acetic. After sonication (20 min, 4 °C), samples were centrifuged for 10 min at 12,000 g and mTHPC fluorescence in the supernatant was measured by spectrofluorometer. mTHPC concentration in tissues was determined using a fluorescence calibration curve obtained from a standard mTHPC solution.

### 2.6. mTHPC Penetration of the 3D Tumor Model

Multicellular tumor spheroids were initiated as previously described [5] by seeding 5 × 10^4^ HT-29 cells/mL in flasks coated with 1% L-agarose. After 3 days, aggregates were transferred into spinner flasks containing 150 mL complete RPMI medium (Integra Biosciences, Zürich, Switzerland) under constant agitation (75 rpm, 37 °C, 5% CO_2_). When spheroids reached 500 µM in diameter, the mTHPC formulations were diluted in RMPI medium supplemented with 2% FBS and added at the concentration of 3.6 µM. After 6 h or 24 h incubation, spheroids were frozen in Tissue-Tek^®^O.C.T^TM^ (ThermoScientific, ThermoFisher, Waltham, MA, USA) and 10 µM thick sections were observed with an epifluorescence microscope (AX-70 Provis, Olympus, Rungis, France) equipped with a 100 W mercury vapor lamp. The filters were set at 400–440 nm band-pass excitation and at 590 nm long-pass emission. Fluorescence images were recorded using × 10 objective and analyzed with the ImageJ 1.51 software (NIH, Bethesda, MD, USA).

### 2.7. Photodynamic Therapy Efficacy

#### 2.7.1. Photodynamic Treatment

When the diameter of tumors reached 4–5 mm (5–9 mice per group), different mTHPC formulations were intravenous injection (i.v.) injected, and 24 h or 6 h after injection, the tumors were irradiated at 652 nm with a diode laser (CeramOptec GmbH, Bonn, Germany) at the fluence rate 100 mW/cm^2^ and fluence of 10 J/cm². The control groups consisted of “drug only” group and “no light, no drug” groups. The diameter of tumors was measured three times per week, and the volume was calculated using the relation V = W² × Y/2, where W and Y are the smaller and larger diameters, respectively. If no sign of tumor recurrence was observed at 90 days post-PDT, it was qualified as tumor cure.

#### 2.7.2. Immunohistochemistry (IHC) for Apoptosis Evaluation

Twenty-four hours after injection, tumors were irradiated (100 mW/cm^2^, 10 J/cm²) as indicated above, and 24 h later mice were sacrificed (4–6 per group). Whole tumors were fixed with 4% formaldehyde for 24 h and further embedded in paraffin to conduct 4-µm-thick sections. Apoptotic cells were evidenced by IHC staining as described earlier [13] with slight modifications. Briefly, sections were subjected to incubation in 10 mM Tris/ethylenediaminetetra-acetic acid (EDTA) solution (pH = 8) at 98 °C for 40 min, with a successive inhibition of endogenous peroxidase activity in a 3% hydrogen peroxide solution for 5 min. After three washings in PBS with Tween^TM^ (PBST), primary antibodies (diluted 1:250) were added for 1 h at room temperature, the samples were washed and biotinylated secondary antibody (diluted 1:200) was applied for another 1 h. Afterwards, the sections were then washed and further incubated in streptavidinperoxidase for 30 min at room temperature. The bound peroxidase was further identified using the NovaRED system and hematoxylin coloration was performed to identify a nuclear counterstaining. The apoptotic cell index in spheroid section was calculated as the number of labeled cells to total number of cells.

### 2.8. Statistics

The data from at least three independent experiments are presented as mean ± standard deviation. The data were evaluated using nonparametric Mann–Whitney’s U test (StatView^TM^ 4.0 software) with a significance level of *p* < 0.05.

## 3. Results

### 3.1. mTHPC-EV Characterization and Behavior in Murine Plasma

**Figure 1:**
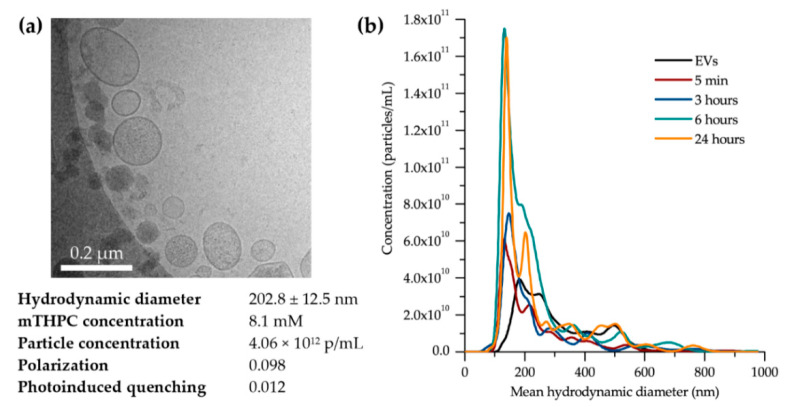
**Characteristics of meta-tetra(hydroxyphenyl)chlorin–extracellular vesicles (mTHPC-EVs).** (**a**) Morphology of mTHPC-EVs determined by cryo-transmission electronic microscopy (TEM); below the scan, the NanoSight photophysical properties of mTHPC-EVs along with the physico-chemical and photophysical characteristics of mTHPC-EVs. (**b**) Incubation kinetics of mTHPC-EVs in the presence of 20% exo-free murine plasma determined by NanoSight.

The size distribution of mTHPC-EVs was analyzed by NTA and confirmed by cryo-TEM, highlighting a mean hydrodynamic diameter at 202.8 ± 12.5 nm and an expected vesicular shape (Figure 1a). After EV isolation, mTHPC concentration, determined spectroscopically, was 8.1 mM for the density of 4.1 × 10^12^ particles/mL. The local concentration of mTHPC in EVs was assessed by measuring the parameters of photoinduced fluorescence quenching and fluorescence polarization of PS. Photoinduced quenching of mTHPC fluorescence in EVs was extremely high (0.012), indicating a high local concentration of mTHPC. mTHPC-EVs demonstrated concentration-induced fluorescence depolarization (0.098) compared to previously shown values for synthetic lipid vesicles (0.32) [14]. Both parameters indicate a high mTHPC loading capacity of EVs.

Similar to a previous study [5], an unexpected behavior of EVs in the presence of 20% exo-free murine plasma (ultracentrifuged murine plasma to remove its EV fraction) was also observed here. In fact, upon the interaction of mTHPC-EVs with serum proteins, the concentration of particles in solution increased with a subsequent decrease in the mean hydrodynamic diameter of the predominant mTHPC-EV population (Figure 1b). After 6 h, the concentration of mTHPC-EVs increased 3.1 times, while the size of particles has been reduced to 144 nm compared to the initial size of 203 nm of the control serum-free plasma (Table 1). In contrast, the NTA of Foslip^®^ showed a constant particle size over incubation (approximately 120 nm) with a gradual decrease in liposome concentration (57% particles after 6 h, Table 1). We previously demonstrated that this decrease was due to membrane destruction of liposomes in the presence of 20% exo-free murine plasma [5].

### 3.2. Plasma Pharmacokinetics of mTHPC-EVs and Foslip^®^

**Figure 2:**
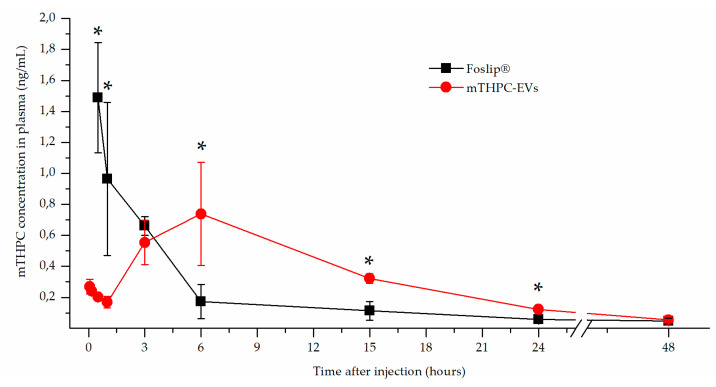
**mTHPC concentration in plasma in function of time after injection.** Intravenous injections of mTHPC-EVs or Foslip^®^ in human colon (HT-29) tumor-bearing mice were realized with 0.30 mg/kg of mTHPC. * *p* < 0.05.

After Foslip^®^ intravenous injection (0.3 mg/kg) to HT-29 tumor-bearing mice, the highest concentration of mTHPC in plasma was detected at 30 min followed by a rapid decrease. Indeed, at 6 h after injection, the concentration of mTHPC in plasma was negligible (less than 0.2 ng/mL). The obtained pharmokinetic profile is in agreement with our previous study, where Foslip^®^ was injected at the concentration of 0.15 mg/kg [15]. Conversely, the distribution profile in plasma after mTHPC-EV i.v. injection at the same mTHPC concentration was very different from that with Foslip^®^. Indeed, as early as 30 min after injection, mTHPC plasma concentration was less than 0.2 ng/mL, while starting from 1 h, mTHPC plasma level was steadily increasing with a maximum peak at 5–7 h (Figure 2).

### 3.3. Biodistribution of mTHPC-EVs and Foslip^®^

**Figure 3:**
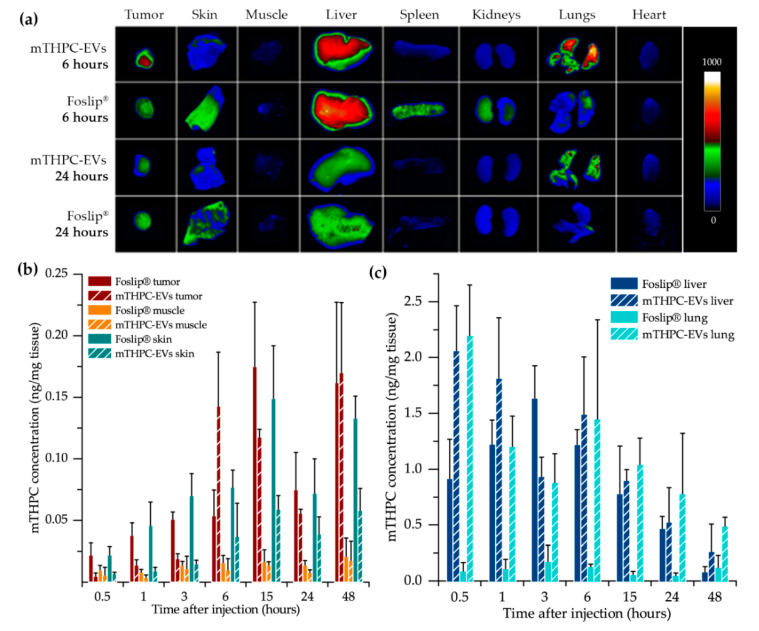
**Kinetics of mTHPC biodistribution in selected organs.** (**a**) Fluorescence imaging was obtained 6 and 24 h after injection of Foslip® or mTHPC-EVs. The concentration of mTHPC in selected tissues, obtained after chemical extraction, is expressed in function of time after intravenous injection: (**b**) tumor, skin and muscle; (**c**) liver and lungs.

Whole-body fluorescence imaging was performed to assess biodistribution kinetics of mTHPC formulations in vivo (data not shown). Based on these data, we selected the most important time intervals and performed ex vivo fluorescence imaging of extracted organs (Figure 3a) with subsequent chemical extraction of mTHPC (Figure 3b,c).

mTHPC levels in tumor, skin and muscle are presented in Figure 3b. The drug concentration in the tumor after mTHPC-EV injection reached a maximum earlier than that with Foslip^®^ (6 h vs. 15 h, respectively). The accumulation of liposomal mTHPC in skin matched the same profile as in the tumor. Importantly, mTHPC concentration in skin 1 h after mTHPC-EV injection was five times lower compared with Foslip^®^ (Figure 3b). This low mTHPC skin concentration slightly increased until 6 h displaying further the plateau (approximately 0.04 ng/ mg tissue), however, it was steadily lower compared with Foslip^®^. mTHPC accumulation in muscle was similar for both formulations and was the lowest compared with other organs (Figure 3b). Negligible fluorescence signal was observed in the heart (Figure 3a). The spleen and kidney accumulation of mTHPC after Foslip^®^ injection peaks at 1 h (data not shown) and 6 h (Figure 3a), respectively, while the levels of mTHPC after mTHPC-EV injection were low at these injection times (Figure 3a). mTHPC concentrations were similar only from 6 h post-injection in the liver for both formulations (Figure 3a,c). A striking difference was observed between mTHPC accumulation in the lungs in function of injected formulation (Figure 3a). Very low levels of Foslip^®^, not exceeding 0.2 ng/mg tissue, were observed during the whole observation period. At the same time, mTHPC-EV accumulation in the lungs was very rapid with a maximum already peaking at 30 min (2.2 ng/mg tissue) and progressively decreasing from 6 h until 48 h (Figure 3c).

**Figure 4:**
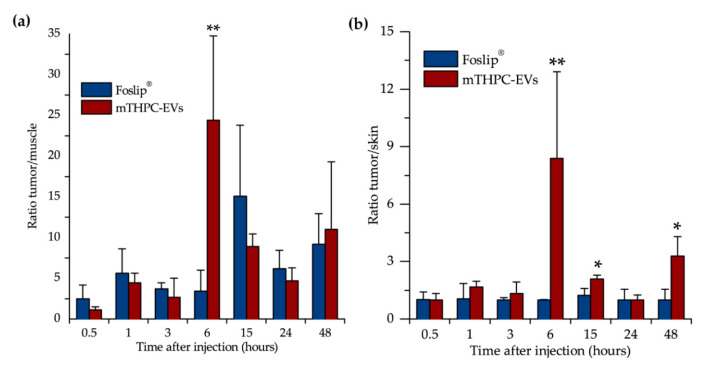
(**a**) Tumor/muscle and (**b**) tumor/skin ratio after Foslip^®^ and mTHPC-EV injection. * *p* < 0.05; ** *p* < 0.01.

The maximum of the tumor/muscle ratio was achieved 6 h after injection of mTHPC-EVs and was five times higher than that of Foslip^®^ (Figure 4a). Likewise, a significantly better tumor/skin ratio was observed for mTHPC-EVs compared with Foslip^®^ at 6 h, 15 h and 48 h after injection (Figure 4b).

### 3.4. Distribution of mTHPC Formulations inside the 3D Tumor Model

**Figure 5:**
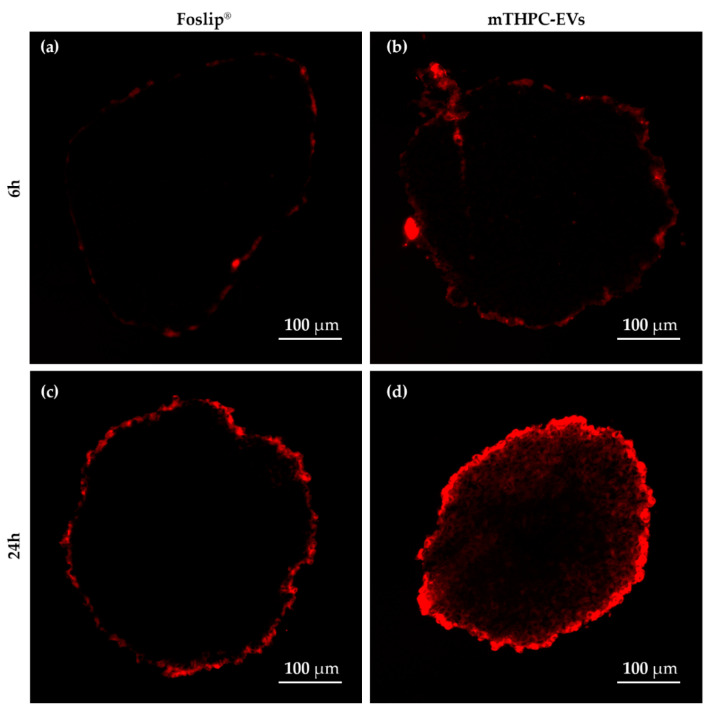
**Fluorescence patterns of HT-29 spheroids after incubation with nanovectorized mTHPC** (3.6 µM). Fluorescence images after 6 h incubation with (**a**) Foslip^®^ and (**c**) mTHPC-EVs and after 24 h of incubation with (**b**) Foslip^®^ and (**d**) mTHPC-EVs.

Fluorescence microscopy was used to evaluate mTHPC diffusion inside 3D multicellular spheroids. The fluorescence of liposomal mTHPC was confined to the spheroid periphery irrespective of incubation time (Figure 5a,c). In contrast, while similar peripheral localization was observed with mTHPC-EVs after 6 h of incubation, strong fluorescence deeper inside the spheroid core (reaching spheroid core) was detected after 24 h incubation (Figure 5b,d).

### 3.5. Photodynamic Efficacy In Vivo

Photodynamic efficacy of Foslip^®^ and mTHPC-EV was tested in HT-29 tumor-bearing mice with a drug-light intervals (DLI) of 6 h and 24 h. The mean tumor growth time is presented in Table 2. No significant difference in tumor response was observed between control non-irradiated groups (PBS, Foslip^®^, mTHPC-EVs), highlighting no toxicity of nanovectorized mTHPC without irradiation. For Foslip^®^/PDT, the mean tumor growth time to reach 10 times the initial tumor volume was similar at 6 h and 24 h DLI and was significantly delayed compared to the control group. In contrast, treatment with mTHPC-EVs/PDT displayed the highest PDT efficacy at 24 h DLI (46.9 days) compared to both 6 h DLI and the control group. In addition, mTHPC-EVs/PDT at 24 h DLI was more effective than that with Foslip^®^ (*p* < 0.01) (Table 2).

**Figure 6:**
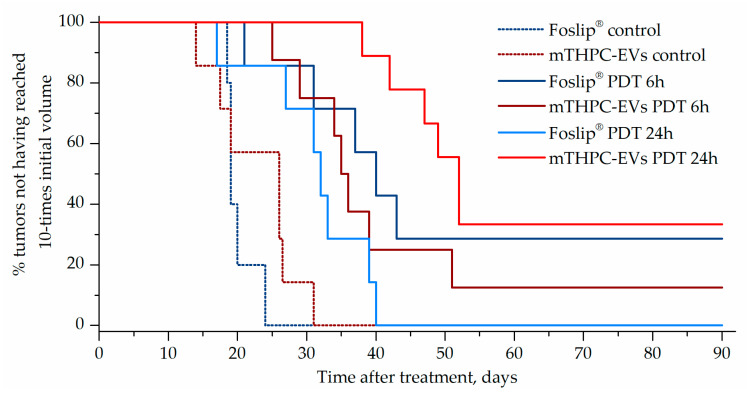
**Kaplan–Meier plots of HT-29 tumor growth delay after PDT with Foslip^®^ and mTHPC-EVs.** Cured mice are included and defined as an absence of tumor regrowth after 90 days post-PDT. The tumor growth of control mice is depicted in the dotted line.

Kaplan–Meier plots of tumor response showed the best antitumor effect in animals after mTHPC-EVs/PDT at a 24 h DLI (33% cured mice), while at a 6h DLI, only 12% of mice were cured. The best effect in Foslip^®^/PDT group was observed at 6 h DLI providing 27% cured animals (Figure 6).

### 3.6. PDT-Induced In Vivo Apoptosis

**Figure 7:**
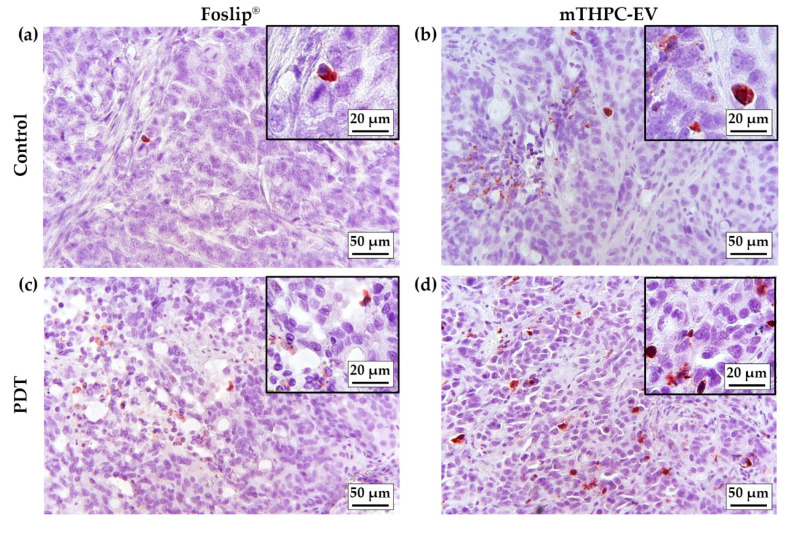
**IHC images of active caspase-3 on paraffin-embedded sections of HT-29 subcutaneous xenografts tumors.** Images were obtained from control tumors injected with (**a**) Foslip^®^ and (**c**) mTHPC-EVs and from irradiated tumors injected with (**b**) Foslip^®^ and (**d**) mTHPC-EVs. Concentration of mTHPC was 0.30 mg/kg.

Apoptotic cells were assessed by IHC staining of active caspase-3. Only a few cells were stained in control samples (Figure 7a,b; Table 3). Maximal staining was obtained in the mTHPC-EVs/PDT group (3.5%) and was found significantly different (*p* < 0.01) from Foslip^®^/PDT (1.56%) (Table 3). A careful examination of immunostaining images showed an increase in apoptotic bodies’ numbers in tissue sections in the irradiated tumor (Figure 7c,d) with typical nuclear morphological changes.

## 4. Discussion

EVs and liposomes share some common structure and constitution features as phospholipid bilayers surrounding an aqueous core, and they are regarded as effective drug nanocarriers. However, EVs as drug delivery systems outperform liposomes, indicating enhanced stability in blood circulation, perfect biocompatibility and a natural ability to target tumors [16]. In fact, EVs naturally contain components that improve cargo delivery to the tumor cells. This was also observed in vivo after systemic EV administration, highlighting an inhibition of tumor growth compared to liposome injection [6].

We have recently demonstrated in an in vitro study considerably improved accumulation and photocytotoxicity of mTHPC-EVs compared with liposomal mTHPC. Moreover, in contrast to Foslip^®^, we noted better stability in plasma with intact membrane integrity [5,15]. We also observed a reduction in the EVs’ size during incubation, from 160 nm at the beginning to 60 nm after 24 h incubation [5]. At this time point (24 h) the concentration of EVs increased three times compared to serum-free samples [5]. In the present study, we observed the same increase in EV concentration (three times) just 6 h after incubation (Figure 1b; Table 1). This more rapid fractionation of EVs is most likely related to the difference in EV production methods between our present research and the previous one. Indeed, turbulence-triggered EV production with the bioreactor used in the present study is based on the physical tension, probably accelerating the interaction of the EVs with plasma components.

After fluorescence imaging acquisition, kinetics of mTHPC biodistribution, following mTHPC-based nanocarrier i.v. injection, were evaluated by chemical extraction (Figure 3). In accordance with literature data, Foslip^®^ reaches a maximal accumulation in the tumor 15 h after injection, followed by a plateau [15]. At the same time, after mTHPC-EV injection, the maximum was already obtained at 6 h followed by a plateau until 48 h (Figure 3b). This typical mTHPC accumulation could be related to the unusual pharmacokinetics of mTHPC-EVs. mTHPC concentration in the plasma for Foslip^®^ was maximal at 30 min after injection and dropped dramatically at 6 h. Indeed, drugs incorporated into the lipid bilayer of liposomes have lower entrapment stability due to the more rapid distribution of the molecule to plasma proteins [6]. The mTHPC plasma concentration profile after mTHPC-EV injection was completely different (Figure 2). At 30 min, the mTHPC plasma level after mTHPC-EV injection was very low, while the maximum mTHPC concentration was observed at 6 h (Figure 2). As demonstrated earlier, this difference could be explained by a rapid capture of EVs by the liver and lungs, just 5 min after i.v. injection, thus considerably diminishing EVs’ circulation time [17]. As anticipated, both nanocarriers were strongly accumulated in the liver [16,18] the organ which is rich in reticulo-endothelial cells (Figure 3a,c). However, a big difference between liposomal and EV formulations is that mTHPC-EVs are strongly accumulated in the lung (five to seven times higher than Foslip^®^, Figure 3c). This result was also demonstrated after the injection of breast cancer-derived EVs, where fluorescence quantification of EVs demonstrated a 13-fold increase compared to liposomes [19]. The authors suggested that this preferential localization is due to the high uptake of EVs by macrophages and surfactant protein C-positive epithelial cells, both strongly present in the lungs. Imai et al. showed colocalization between B16BL6 EVs and endothelial cells in the lung [20]. The biodistribution of EVs in vivo is affected by routes of injection and cellular origin of EVs [21], therefore, we can suppose that endothelial-derived EVs have a preferential lung accumulation due to intrinsic tropism.

We also performed pharmacokinetic measurements with mTHPC-loaded EVs derived from mesenchymal stem cells and observed that the pharmacokinetic plasma profile is similar to that in the present study (data not shown). Interestingly, stem cells also display similar pharmacokinetics after systemic administration, being first physically trapped in the lung capillary network and subsequently co-localizing to the disease site [18]. This similarity allows us to generalize the intrinsic properties of EVs and cells in relation to these unusual pharmacokinetics compared to liposomes. Pharmacokinetics in our study were analyzed via drug extraction. Therefore, either EV entrapment in liver/lungs capillaries may be reverted, leading to mTHPC-EV recirculation in the bloodstream, or free mTHPC may be released from EVs in the blood. Both processes may also take place concurrently. A similar pharmacokinetic profile was earlier reported for free mTHPC in sheep and human plasma [22].

Maximal mTHPC–EV accumulation in the tumor was found at 6 h and was similar to that of Fospeg^®^ (liposomal mTHPC coated with polyethylene glycol (PEG)) [15]. This similarity could be explained by stealth properties of PEGylated nanocarriers. Indeed, PEG is generally added on nanocarriers to inhibit their recognition and uptake by the reticuloendothelial system, while EVs could naturally escape the immune system recognition [23]. In addition, compared with Foslip^®^, mTHPC-EV showed lower drug accumulation in the skin for all injection times (Figure 3b), thus indicating less severe side effects after PDT application.

In order to predict in vivo therapeutic efficacy, we used a 3D model of HT-29 multicellular spheroids. In contrast to monolayer cells, this model mimics cellular communication and cell–matrix interactions and represents the microenvironment encountered by the nanocarrier after extravasation from tumor blood vessels [24]. In addition, it was previously demonstrated that a relationship exists between drug diffusion, its accumulation inside spheroid, and therapeutic efficacy [25]. This phenomenon has already been demonstrated with doxorubicin nanocarriers. The drug was confined to the spheroid periphery and was restricted to the vascular region in vivo, which was detrimental for the cytotoxic activity [26].

In our recent study [5], we demonstrated a deep penetration of mTHPC-EVs through spheroids. In the present study we used different production method of EVs (turbulence-triggered production with a bioreactor), but a similar penetration pattern of mTHPC-EVs in spheroids 24h after incubation was confirmed in the present work (Figure 5). It was also shown that cholesterol-free and 1,2-dioleoyl-3-trimethylammonium-propane (DOTAP) liposomes, such as Foslip^®^, did not penetrate the spheroids more than 40 µm in depth [27]. Meanwhile, the composition of the EV lipid membrane provides flexibility and stabilizes EV vesicles, thus increasing the probability of penetrating the tumor tissue.

We also investigated therapeutic efficacy in vivo. At 24 h after injection with mTHPC-EVs, a delay of 47 days was necessary to achieve ten times the initial volume of the tumor, compared to 31 days with Foslip^®^ (Table 2). Additionally, 33.3% of tumor remission was observed 90 days after PDT (Figure 6). We next investigated therapeutic efficacy in vivo and its correlation with maximal tumor drug concentration kinetics and maximal tumor penetration kinetics. Based on the deep mTHPC-EV penetration of spheroids at 24 h post-incubation, we can suggest an improved intratumor mTHPC-EV penetration in vivo, thus explaining a better anti-tumor efficacy of mTHPC-EVs. In contrast, at 6 h, when the intratumoral concentration of mTHPC-EVs was maximal (and mainly peripheral), no significant difference was observed between both formulations, neither in mTHPC diffusion across the spheroids nor in vivo PDT efficacy (Figure 3, Table 2). Thus we can suggest that PS penetration, rather than tumor drug concentration, is a predominant factor for therapeutic efficacy.

Apoptosis in 3D cells demonstrated a high, albeit not significant, number of caspase-3 positive cells in both mTHPC-EV- and Foslip^®^-treated spheroids (ca. 30%) [5]. In contrast, in an in vivo study, better PDT efficacy was not conditioned by the induction of apoptosis. In fact, only 1.6% and 3.5% of apoptotic cells were induced by Foslip^®^/PDT and mTHPC-EVs/PDT, respectively (Table 3). Low levels of apoptotic cells (2.6%), despite efficient PDT, were also observed in Foslip^®^-mediated PDT in the osteosarcoma mouse model [28].

## 5. Conclusions

Cell-derived vesicles possess some remarkable properties, rendering them attractive for the delivery of anticancer drugs. Similar to liposomes, EVs are made up of a phospholipid bilayer surrounding an aqueous core. Our present study clearly demonstrated an advantage of mTHPC-EVs over liposomal mTHPC formulations in terms of stability in plasma, mTHPC diffusion across microtumor models and PDT efficacy in vivo. PDT treatment with EVs resulted in 33% of tumor remission in xenografted mice at 24 h DLI, while no remission was detected after Foslip^®^–PDT under the same experimental conditions. The main determinant factor responsible for this high mTHPC-EVs/PDT efficiency could be a deep mTHPC intratumor penetration as suggested by a significantly homogenous distribution of mTHPC-EVs across a 3D tumor model when compared with Foslip^®^. Our ongoing studies assess intratumor mTHPC distribution in different nanoformulations in sophisticated 3D platforms and xenografted tumor-bearing rodents.

## 6. Patents

Patent WO/2020/136361 (2020). Piffoux, M.; Millard, M.; Grangier, A.; Marangon, I.; Bolotine, L.; Wilhelm, C.; Gazeau, F.; Silva, A. “A fluid system for producing extracellular vesicles comprising a therapeutic or imaging agent and associated method”.

## Figures and Tables

**Table 1:** The behavior of mTHPC-EVs and Foslip^®^ exo-free murine plasma determined by NanoSight.

Nanocarriers	Characteristic	Without Serum	1 h	3 h	6 h	24 h
**mTHPC-EVs**	Concentration, × 10^12^ p/mL	4.16 (100%)	6.49 (156%)	7.77 (187%)	12.7 (314%)	8.73 (215%)
	Mean size of major peak, nm	203	177.2	161.5	144.5	151.4
**Foslip^®^**	Concentration, × 10^12^ p/mL	3.4 (100%)	2.7 (77%)	2.07 (61%)	1.99 (57%)	1.01 (29%)
	Mean size of major peak, nm	124.8	123.8	117.4	119	116.1

**Table 2:** Remission occurrence and number of days after treatment required to reach 10 times the initial tumor volume, excluding remission cases. Remission was defined as the absence of tumor regrowth at 90 days after treatment. ^a^: *p* < 0.05, ^A^: *p* < 0.01, statistically different from control; ^b^: *p* < 0.01, statistically different between Foslip^®^ and mTHPC-EVs. Non-irradiated tumors were considered as the control group (drug, no light). *n* = 5–7 mice for control; *n* = 7–9 mice for photodynamic therapy (PDT) conditions.

Drug-Light Intervals	Number of Days after Buffer i.v.	Number of Days after Foslip^®^ i.v.	Cured Mice	Number of Days after mTHPC-EV i.v.	Cured Mice
Control	22.5 ± 3.5	20.1 ± 2.2	0/7	22.8 ± 6.1	0/7
6 h		34.1 ± 8.6 ^a^	2/7	35.7 ± 8.2 ^a^	1/8
24 h		30.8 ± 7.3 ^a^	0/8	46.9 ± 5.7 ^A,b^	3/9

**Table 3:** **Apoptotic index calculated from the quantification of apoptotic cells.** The apoptotic index was determined from at least 1000 tumor cells in each section. *n* = 4 mice per condition. Non-irradiated tumors were selected as the control group (drug, no light). ^a^: *p* < 0.05, statistically different from control; ^b^: *p* < 0.01, statistically different between Foslip^®^ and mTHPC-EVs.

Irradiation Condition	Foslip^®^	mTHPC-EVs
No Light	0.70 ± 0.002	0.69 ± 0.001
PDT (24 h DLI)	1.56 ± 0.001	3.49 ± 0.009 ^a,b^
